# Data for the inhibition effects of recombinant lamprey CRBGP on the tube formation of HUVECs and new blood vessel generation in CAM models

**DOI:** 10.1016/j.dib.2016.01.004

**Published:** 2016-01-13

**Authors:** Qi Jiang, Yu Liu, Meng Gou, Jianmei Han, Jihong Wang, Qingwei Li, Rong Xiao

**Affiliations:** a School of Life Sciences, Liaoning Normal University, Dalian 116081, PR China; b Lamprey Research Center, Liaoning Normal University, Dalian 116081, PR China

**Keywords:** Lamprey, CRISP, rL-CRBGP, Anti-angiogenic activity

## Abstract

In the present data article, lamprey cysteine-rich buccal gland protein (CRBGP) which belongs to cysteine-rich secretory proteins (CRISPs) family was recombinant and expressed in *Rosetta blue* cells. After identification, the recombinant protein was purified through affinity chromatograph. The inhibition effects of recombinant lamprey CRBGP (rL-CRBGP) on tube formation of human umbilical vein endothelial cells (HUVECs) and new blood vessel generation in chick chorioallantoic membrane (CAM) models were analyzed. This paper contains data related to research concurrently published in “Anti-angiogenic activities of CRBGP from buccal glands of lampreys (*Lampetra japonica*)” [1].

## 1 **Specifications table**

**Table:** 

Subject area	*Biology*
More specific subject area	*Biochemistry*
Type of data	*Figure*
How data was acquired	*Microscope, mass spectroscopy, camera*
Data format	*Raw and analyzed, etc.*
Experimental factors	*PBS and rL-CRBGP were added in HUVECs and CAM models*
Experimental features	*Protein recombination, expression, separation, purification and identification. Cell culture, tube formation and CAM model assay*
Data source location	*Dalian, China*
Data accessibility	*Data is with this article*

## 2 **Value of the data**

• These data are valuable for the soluble expression of the other CRISP family members.
• These data are valuable for the studies of the relationship between other CRISP family members and angiogenesis.

## 3 Data

**Fig. 1:**
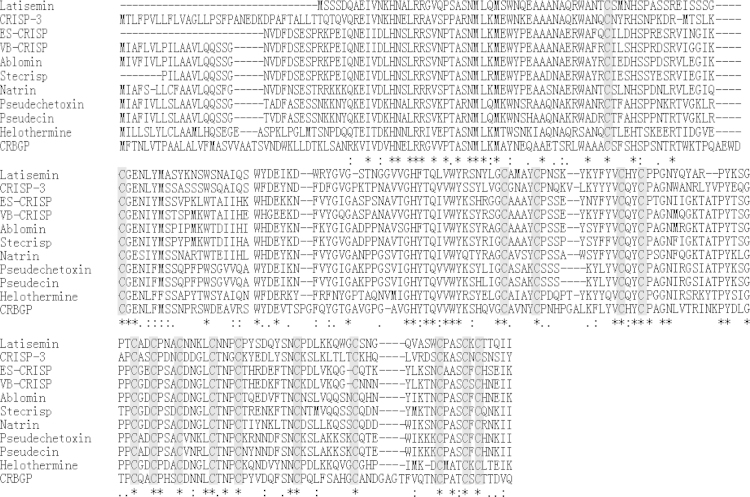
Multiple sequence alignments of lamprey CRBGP with CRISPs from the other species by using ClustalX (1.81). The accession numbers of the amino acid sequences extracted from the ExPASy database are as follows: Latisemin, *Larimichthys crocea*, 808871467; CRISP-3, *Homo sapiens*, 1706135; ES-CRISP, *Echis carinatus sochureki*, sequence was extracted from Ref. [4]; VB-CRISP, *Vipera berus*, 487523159; Ablomin, *Gloydius blomhoffii*, 21305551; Stecrisp, *Trimeresurus stejnegeri*, 45476808; Natrin, *Naja atra*, 32492059; Pseudechetoxin, *Pseudechis australis*, 23264042; Pseudecin, *Pseudechis porphyriacus*, 23264044; Helothermine, *Heloderma horridum*, 606921; CRBGP, *Lethenteron camtschaticum* (*Lampetra japonica*), 145046200. Dashes represent gaps inserted into the alignment. Identical residues are indicated by asterisks. Strong and weak homologous residues are indicated in colons (:) and dots (.), respectively. The conserved cysteine residues are covered with gray boxes.

**Fig. 2:**
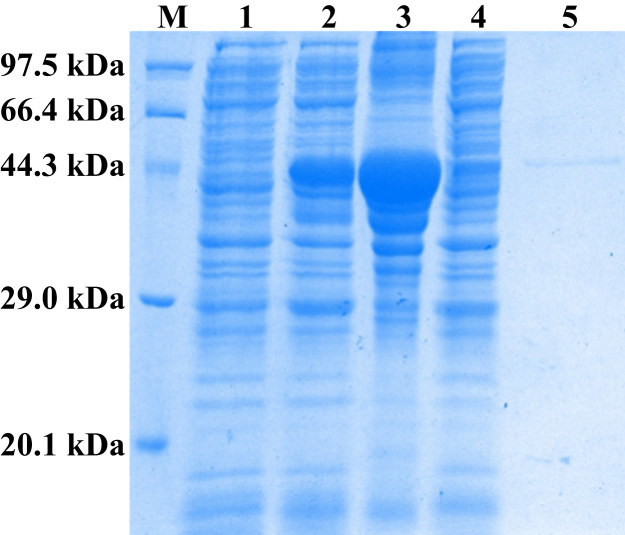
The expression and purification of rL-CRBGP. The rL-CRBGP was separated by 12% sodium dodecyl sulfate-polyacrylamide gel electrophoresis (SDS-PAGE) and stained with Coomassie brilliant blue R-250. Lane M, low molecular weight protein marker; lane 1, crude lysate of *Rosetta blue* cells before IPTG induction (28 μg); lane 2, crude lysate of *Rosetta blue* cells after induction with 1 mM IPTG for 36 h (30 μg); lane 3, precipitation from the induced *Rosetta blue* cells after sonication on ice for 1 h (33 μg); lane 4, supernatant from the inducted *Rosetta blue* cells after sonication on ice for 1 h (28 μg); lane 5, the purified rL-CRBGP (7.5 μg).

**Fig. 3:**
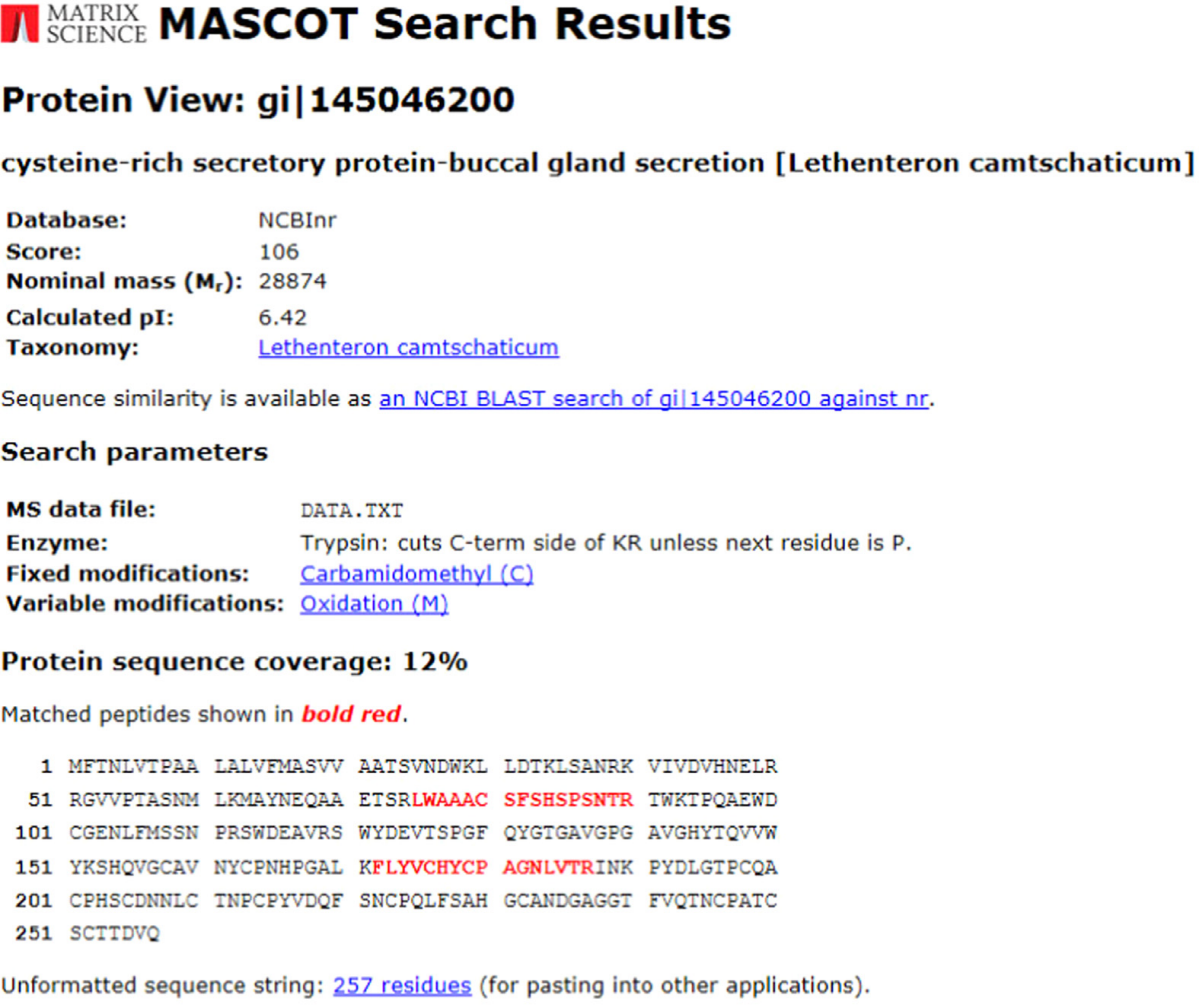
The recombinant protein was identified as rL-CRBGP by MALDI-TOF/TOF analysis.

**Fig. 4:**
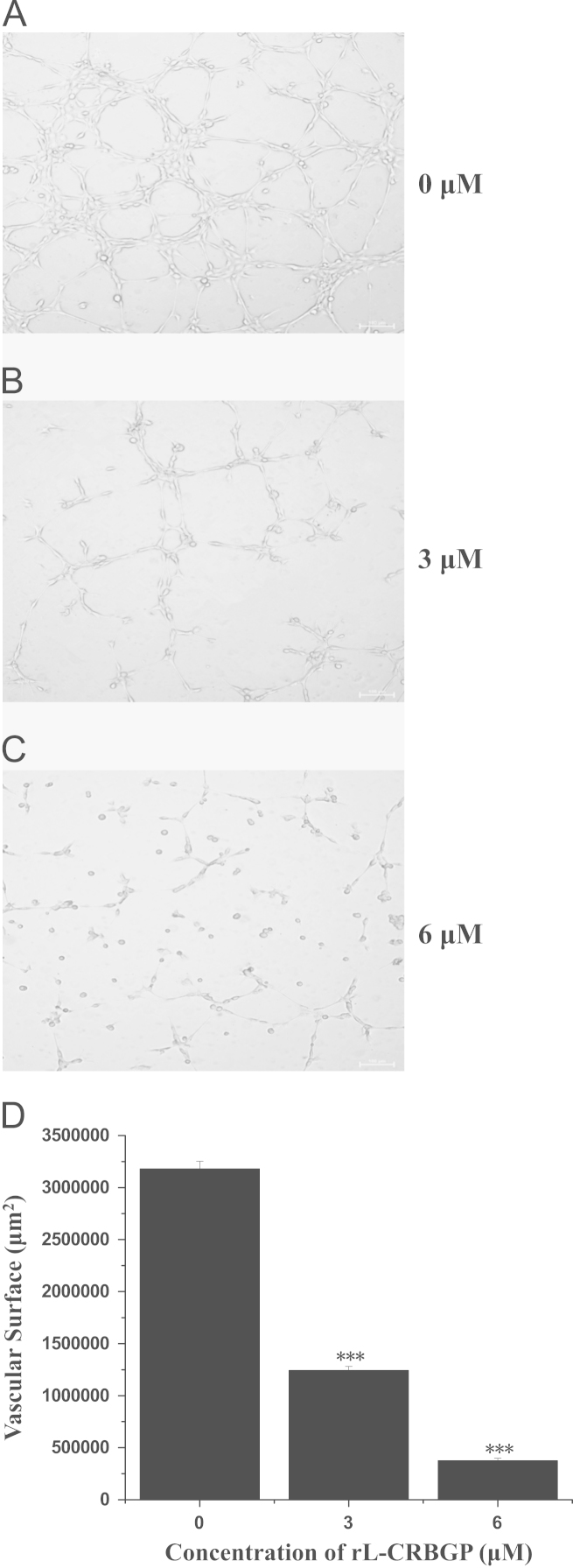
rL-CRBGP inhibited tube formation of HUVECs. Similar to the tube formation assay of native lamprey CRBGP [1], HUVECs were also treated with PBS (panel A), 3 μM (panel B) or 6 μM (panel C) rL-CRBGP in the Matrigel-coated 24-well plates at 37 ˚C for 16 h. After capturing the morphology of capillary-like tube through a microscope (Nikon, Japan), the vascular surface of formed tubes per microscopic observation field was counted and shown in panel D. The significant differences between the PBS and the rL-CRBGP treated groups were indicated with asterisks (***P<0.001).

**Fig. 5:**
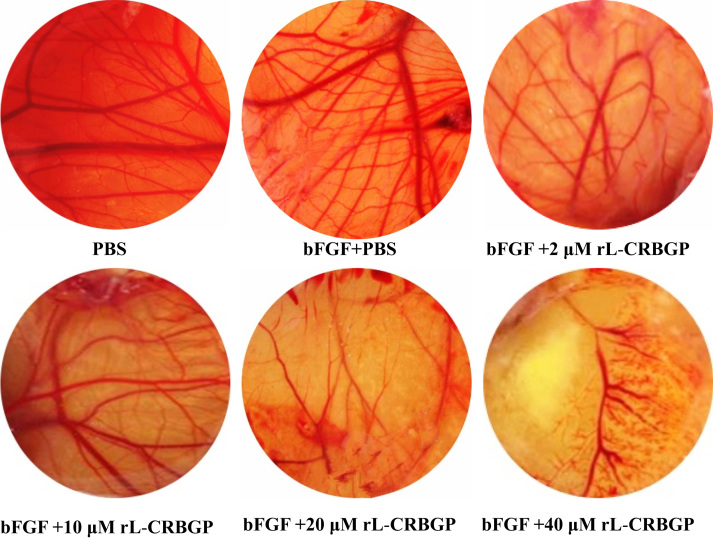
rL-CRBGP inhibited basic fibroblast growth factor (bFGF)-induced angiogenesis in CAM assays. Similar to the anti-angiogenic assay of native lamprey CRBGP in CAM models, PBS (control) and rL-CRBGP (2, 10, 20 and 40 μM) were also added onto the methylcellulose disc implanted into the bFGF-induced chick embryos at 37 ˚C for 24 h, respectively. When the methylcellulose disc was removed, the chick embryos were captured by digital camera (Nikon, Japan).

As shown in Fig. 1, lamprey CRBGP is a very conservative gene and has 45% sequence identity with the ES-CRISP from the snake venom of *Echis carinatus sochureki*. Subsequently, lamprey CRBGP was subcloned into a pEGX-4T-1 vector and expressed as a Glutathione S-transferase (GST)-tagged fusion protein in *Rosetta blue* cells with the molecular weight of 51.6 kDa (Fig. 2). After identification by matrix-assisted laser desorption/ionization time of flight (MALDI-TOF/TOF) analysis, rL-CRBGP was found to exhibit the anti-angiogenic activities in both tube formation and CAM assays (Fig. 3, Fig. 4, Fig. 5).

## 4 Experimental design, materials and methods

### 4.1 Sequence alignment

Additional 10 CRISP sequences from the other species were obtained from ExPASy (http://www.expasy.ch/tools/blast). The multiple sequence alignments of CRISPs were performed by ClustalX (1.81) software using default settings [2].

### 4.2 Expression, purification, and identification of rL-CRBGP

A pair of PCR primers (CRBGP-F: 5′-CCGGAATTCGCGAGCGTCGTGGCGGCGACA-3′; CRBGP-R: 5′-AGAAGAATGCGGCCGCCTGCACATCCGTCG-3′) was designed based on the sequence of lamprey CRBGP [3], flanked by an *Eco*R I and a *Not* I restriction site. Lamprey CRBGP was amplified and subcloned into a pEGX-4T-1 vector with a GST-tag. rL-CRBGP was expressed in *Rosetta blue* cells induced with 1 mM isopropyl-1-thio-β-D-galactopyranoside (IPTG) for 36 h. The cells were collected by centrifugation, and washed in PBS for twice (pH 7.4). Subsequently, the cells were resuspended in the PBS (pH 7.4) and sonicated on ice for 60 min. After centrifugation, the soluble supernatant was collected and subjected to a GSTrap^TM^ 4B column (GE, USA) equilibrated with binding buffer (PBS, pH 7.4). After washing the column with wash buffer (PBS, pH 7.4), the rL-CRBGP was collected in elution buffer containing 50 mM Tris–HCl, 20 mM glutathione (pH 8.0). The concentration of rL-CRBGP was also measured using a bicinchoninic acid (BCA) protein assay Kit (Beyotime Biotechnology, China). The purified rL-CRBGP was analyzed by 12% SDS-PAGE and stained with Coomassie brilliant blue R-250. The rL-CRBGP was digested with trypsin (25 mM, Promega) in-gel overnight and identified by MALDI-TOF/TOF mass spectrometry (Bruker, USA).

### 4.3 Anti-angiogenic activity assay of rL-CRBGP

Similar to that of native lamprey CRBGP, the anti-angiogenic activity of rL-CRBGP was also performed in both tube formation assay and CAM models according to the methods reported by Qi Jiang and colleagues [1].
