# Supplementary material for: Data for the inhibition effects of recombinant lamprey CRBGP on the tube formation of HUVECs and new blood vessel generation in CAM models
Source: Data Brief. 2016 Jan 13;6:661–7. doi: 10.1016/j.dib.2016.01.004 (PMC4735471; doi:10.1016/j.dib.2016.01.004)
Supplement: Supplementary file 1 — Supplementary material [file mmc1.docx]

Conflict of interest form

Dear Editors:

The authors claim that we do not have any conflict of interest and none of the material in the paper has been published or is under consideration for publication elsewhere.

Qi Jiang

Yu Liu

Meng Gou

Jianmei Han

Jihong Wang

Qingwei Li

Rong Xiao
